# Simultaneous targeting of glycolysis and oxidative phosphorylation as a therapeutic strategy to treat diffuse large B-cell lymphoma

**DOI:** 10.1038/s41416-022-01848-w

**Published:** 2022-05-26

**Authors:** Richard A. Noble, Huw Thomas, Yan Zhao, Lili Herendi, Rachel Howarth, Ilaria Dragoni, Hector C. Keun, Christopher P. Vellano, Joseph R. Marszalek, Stephen R. Wedge

**Affiliations:** 1 Cancer Research Horizons Therapeutic Innovation, Translational and Clinical Research Institute, Newcastle University, Newcastle upon Tyne, UK; 2 Department of Surgery & Cancer and Department of Metabolism, Digestion & Reproduction, Imperial College London, London, UK; 3 Newcastle University Centre for Cancer, Newcastle University, Newcastle upon Tyne, UK; 4 Centre for Drug Development, Cancer Research UK, London, UK; 5 TRACTION Platform, Therapeutics Discovery Division, University of Texas M. D. Anderson Cancer Center, Houston, TX USA

**Keywords:** Pharmacology, Cancer metabolism, B-cell lymphoma

## Abstract

**Background:**

We evaluated the therapeutic potential of combining the monocarboxylate transporter 1 (MCT1) inhibitor AZD3965 with the mitochondrial respiratory Complex I inhibitor IACS-010759, for the treatment of diffuse large B-cell lymphoma (DLBCL), a potential clinically actionable strategy to target tumour metabolism.

**Methods:**

AZD3965 and IACS-010759 sensitivity were determined in DLBCL cell lines and tumour xenograft models. Lactate concentrations, oxygen consumption rate and metabolomics were examined as mechanistic endpoints. In vivo plasma concentrations of IACS-010759 in mice were determined by LC-MS to select a dose that reflected clinically attainable concentrations.

**Results:**

In vitro, the combination of AZD3965 and IACS-010759 is synergistic and induces DLBCL cell death, whereas monotherapy treatments induce a cytostatic response. Significant anti-tumour activity was evident in Toledo and Farage models when the two inhibitors were administered concurrently despite limited or no effect on the growth of DLBCL xenografts as monotherapies.

**Conclusions:**

This is the first study to examine a combination of two distinct approaches to targeting tumour metabolism in DLBCL xenografts. Whilst nanomolar concentrations of either AZD3965 or IACS-010759 monotherapy demonstrate anti-proliferative activity against DLBCL cell lines in vitro, appreciable clinical activity in DLBCL patients may only be realised through their combined use.

## Background

A wide range of metabolically targeted agents are currently under clinical investigation including examples targeting nucleotide synthesis, amino acid metabolism and the TCA cycle [1]. Despite this interest, metabolic heterogeneity both within and around tumours means implementing these inhibitors in the correct context and with suitable combinatorial partners will be crucial for these agents to be effective [2–5].

One approach to exploit the metabolic vulnerability of tumour cells has been through the development of AZD3965, an orally bioavailable inhibitor of monocarboxylate transporter 1 (MCT1), which is currently under Phase I clinical investigation (NCT01791595). Recent studies have demonstrated that AZD3965 and structurally related MCT1 inhibitors can prevent lactate efflux in highly glycolytic tumour types that lack MCT4, inducing a feedback inhibition of glycolytic flux that can impart a significant anti-proliferative effect [6, 7]. MCT1 expression has also been shown to have prognostic relevance in several cancer types [8–10]. We focussed our study on DLBCL, the most prevalent form of non-Hodgkin lymphoma that, whilst generally responding well to first-line treatment with chemotherapy and rituximab, represents a significant unmet need in patients with relapsed or refractory disease for which limited therapeutic options exist [11]. Clinically, nearly three-quarters of DLBCL cases have been found to express MCT1 but not MCT4 suggesting that they may be responsive to MCT1 inhibition [12, 13].

An alternative approach to targeting tumour metabolism is through inhibition of Complex I of the mitochondrial electron transport chain (NADH-ubiquinone oxidoreductase), which plays an essential role in oxidative phosphorylation (OXPHOS) but also biosynthesis, redox control during tumour cell proliferation, resistance to cell death, and metastasis [14]. Several small-molecule inhibitors of Complex I have been identified as potential anticancer agents [15, 16]. This also includes a highly potent and selective small-molecule Complex I inhibitor, IACS-010759, described recently, that is under clinical evaluation (NCT 02882321) [17]. IACS-010759 has been demonstrated to be efficacious in models of acute myeloid leukaemia, mantle cell lymphoma and a range of solid tumours [17, 18]. Such an inhibitor may also be relevant to the treatment of “OxPhos-DLBCL” tumours; a subset defined on the basis of having an elevated expression of electron transport chain genes and which demonstrate an increased reliance upon mitochondrial energy production [19].

As metabolic pathways are necessarily adaptable and interlinked to deal with changing nutrient and oxygen availability it may be necessary to utilise combination approaches in order to limit compensatory changes and enhance activity [20]. In cells sensitive to AZD3965, the feedback inhibition of glycolysis can result in a greater reliance on OXPHOS for ATP generation [12]. Conversely, IACS-010759 treatment has been found to increase sensitivity to the inhibition of phosphogluconate dehydrogenase or lactate dehydrogenase, suggesting that it can induce a greater reliance on glycolysis in certain cell types [21, 22]. Simultaneous inhibition of both MCT1 and Complex I could therefore represent a promising strategy for the targeted treatment of DLBCL tumours that lack MCT4.

Our data indicate that the combination of AZD3965 and IACS-010759 can be synergistic in a range of MCT1+/MCT4− DLBCL cell lines, resulting in tumour cell death. A combination effect can also be observed in vivo with plasma concentrations of IACS-010759 that are comparable to those achieved in patients. Collectively these studies provide a rationale for combining AZD3965 and IACS-010759 treatment for the clinical management of DLBCL.

## Methods

### Cell lines, compounds and antibodies

Farage, Pfeiffer and Toledo were obtained from ATCC and BJAB, NUDHL1, OCILY-19, RIVA and WSU-DLCL2 were obtained from DSMZ. OCILY-3 were provided as a kind gift from Prof. Martin Dyer. Cell lines were authenticated by short tandem repeat (STR) analysis (New Gene). All cell lines were cultured in RPMI-1640 media (Sigma) supplemented with 10% foetal calf serum (Gibco) and grown at 37 °C under 5% CO_2_ and routinely tested for mycoplasma contamination.

Where available, the cell of origin (COO) subtype [23, 24] and consensus cluster classification (CCC) [25–27] of DLBCL cell lines were assigned from the literature. Details of genetic alterations in cell lines sourced from CCLE [28] were accessed from cBioPortal [29].

For in vitro studies AZD3965 (AstraZeneca) and IACS-010759 (MD Anderson Cancer Center) were prepared as 10 mM stock solutions in dimethyl sulfoxide (DMSO). For in vivo studies, AZD3965 and IACS-010759 were formulated as previously described [8, 17]. Western blotting was conducted using antibodies to MCT1 (20139-1-AP, Proteintech), MCT4 (22787-1-AP, Proteintech) and GAPDH (sc-47724, Santa Cruz).

### Mitochondrial respiration measurements

Metabolimetry experiments were performed using the XF-96 Analyzer apparatus from Seahorse Bioscience (FCCF Newcastle University). Cells were attached to CellTak (Corning) coated plates at 100,000–150,000 cells per well on the day of the experiment in DMEM with 11 mM glucose, 1 mM pyruvate and 2 mM glutamine (no sodium bicarbonate) as previously described [12]. For the mitochondrial stress test, oligomycin (1 µg/ml), trifluoromethoxy carbonylcyanide phenylhydrazone (FCCP 1–1.5 μM), and a mixture of antimycin (2.5 µg/ml) and rotenone (0.5 μM) were used. OCR values were used to calculate basal respiration rates following treatment with IACS-010759.

### In vivo studies

Toledo, RIVA (1 × 10^7^) and Farage (5 × 10^6^–1 × 10^7^) cells in 50% matrigel were injected subcutaneously into the flanks of female NSG™ (NOD.Cg-*Prkdc*^*scid*^ *IL2rg*^*tm1Wjl*^/SzJ) mice at 8–10 weeks of age (Charles River). Tumour diameter and volume were calculated based on calliper measurements of tumour length and height using the formula tumour volume = (length × width^2^)/2. When tumours reached approximately 200 mm^3^, mice were randomised into control and experimental groups. Researchers were not blinded to treatment group allocations. AZD3965 (100 mg/kg, BID 8 h apart) and/or IACS-010759 (0.25–7.5 mg/kg, QD 2 h after the first dose of AZD3965) and vehicle controls were administered by oral gavage. Combination experiments were vehicle-controlled throughout (i.e. mice receiving AZD3965 alone also received the IACS-010759 vehicle, and vice versa) so that an equivalent number of oral gavages were administered to all mice via an identical schedule. The number of days for each individual tumour to quadruple in size from the start of the treatment (RTV4) was calculated for the individual tumours in each group and used as the endpoint for efficacy studies.

Hypoxia marker pimonidazole (60 mg/kg) was administered by intraperitoneal injection 1.5 h prior to excising tumours. Samples were formalin-fixed paraffin-embedded (FFPE) using standard methods. Regions of hypoxia were detected via IHC using the hypoxyprobe antibody (Natural Pharmacia International).

All in vivo experiments were reviewed and approved by the Newcastle University Animal Welfare and ethical review body, and performed according to guidelines set out by an ad hoc committee of the National Cancer Research Institute and national law [30].

### Pharmacokinetic studies

Female 8–10-week-old NSG mice (Charles River) were treated with IACS-010759 p.o. (0.5, 1 or 5 mg/kg). At selected time points animals were bled by either tail vein puncture or cardiac puncture under terminal anaesthesia at selected time points post-treatment (0.25–24 h, 3 mice/timepoint). Blood was collected into heparinised tubes, and plasma was separated and stored at –20 °C until analysed.

Drug concentrations were determined by LC-MS calibrated with standards prepared in control plasma. Pharmacokinetic parameters were determined using a non-compartmental (trapezoidal) method. The terminal half-life was determined using log-linear regression of the final time points.

### Determination of intracellular lactate, protein, cell growth and viability

Lactate concentration was determined by luminescent assay (Promega) and normalised to protein content. For growth inhibition assays, cells were plated overnight before treatment for 72 h and assessed using an XTT assay (Sigma). GI_50_ values were determined using GraphPad Prism (version 9). Cell number and viability were determined concurrently after 72 h of AZD3965 ± IACS-010759 treatment using a hemocytometer and trypan blue exclusion, respectively. Synergy was assessed using the Zero Interaction Potency (ZIP) method with SynergyFinder 2.0 [31]. Each plot represents a single analysis performed on mean viability data taken from 4 to 5 independent experiments.

### Metabolomics

The ex vivo intracellular metabolite composition of tumour xenograft samples was assessed in samples snap-frozen 6 h following a single dose of AZD3965 (100 mg/kg), IACS-010759 (4 h, 0.5 mg/kg) or a combination regimen of AZD3965 followed by IACS-010759. Metabolomic analysis was performed via GC-MS as previously described [12]. Pathway enrichment analysis was performed with lists of significantly altered metabolites against pathway-associated metabolite sets from SMPDB (version 2.0) [32] using Metaboanalyst (version 4.0) [33].

### Immunohistochemistry

DLBCL xenograft tissues were fixed in 10% formalin solution, processed, and embedded in paraffin. Hypoxyprobe scoring of pixel or nuclear positivity was performed using the Aperio Scanscope Console (Leica Biosystems) and the images were analysed using ImageScope Nuclear v9 algorithm to report the total number of nuclei counted and the percentage of positive cell nuclei.

### Statistics

Statistical significance between two treatment groups was examined using a two-tailed Student’s *t*-test unless otherwise stated in the figure legend. Statistical differences in time to reach RTV4 were assessed using the Kaplan–Meier method and Log-rank (Mantel–Cox) test. *P* values ≤0.05 were considered significant and signified as follows *P < 0.05, **P < 0.01, ***P < 0.001. NS indicates not significant, *P* > 0.05. All statistical analyses were performed using Graphpad Prism (version 9) or SPSS Statistics (version 26).

## Results

### The combination of AZD3965 and IACS-010759 induces cell death in DLBCL cell lines lacking MCT4

**Fig. 1:**
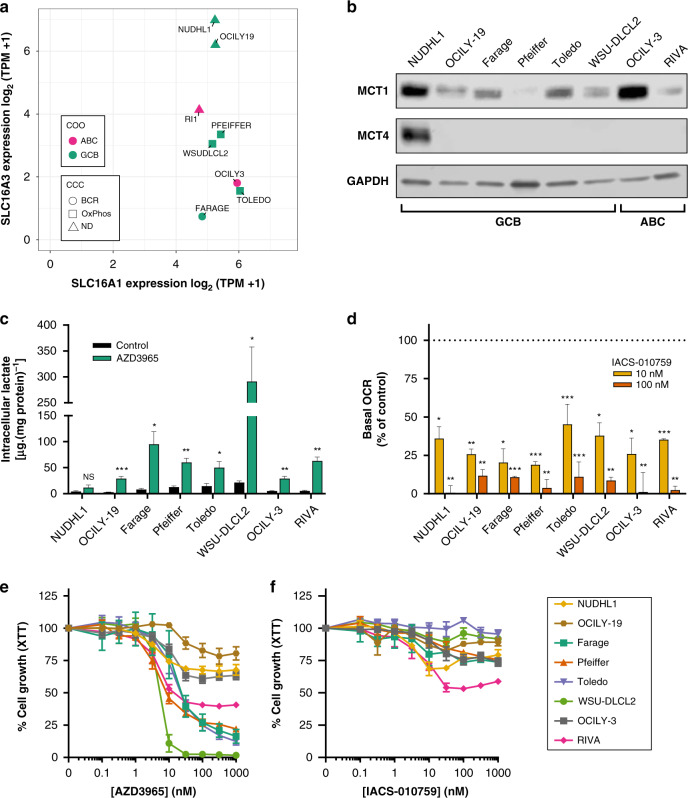
In vitro monotherapy responses to AZD3965 and IACS-010759 in DLBCL. **a** *SLC16A1* (*MCT1*) and *SLC16A3* (*MCT4*) RNA expression (log_2_[transcripts per million + 1]) in the cell panel examined. **b** MCT1 and MCT4 protein expression in cell lines using GAPDH as a loading control. The cell of origin (COO) subtype, either germinal centre B-cell (GCB) or activated B-cell (ABC), is indicated. **c** Concentration of intracellular lactate in DLBCL cell lines treated for 24 h with AZD3965 (10 nM) (mean + SEM; *n* = 3). **d** Dose-dependent changes in oxygen consumption rate in DLBCL cells 24 h following pre-treatment with IACS-010759 (mean + SD; *n* = 2). **e** Sensitivity of DLBCL cells to 72 h single-agent treatment with the MCT1 inhibitor, AZD3965 or **f**, Complex I inhibitor, IACS-010759, assessed by XTT assay (mean ± SEM; *n* = 3–4).

Farage, Pfeiffer, RIVA, Toledo and WSU-DLCL2 DLBCL cell lines were selected based on their previously defined MCT1+/MCT4− protein expression and known sensitivity to AZD3965 [6, 12]. OCILY-3 was included as a previously identified AZD3965-resistant DLBCL cell line [6]. NUDHL1 and OCILY-19 DLBCL cells were also included as they express comparatively high levels of *SLC16A3* (MCT4 gene) mRNA (Supplementary Fig. S1a), and were therefore considered likely to demonstrate resistance to MCT1 inhibition. That the expression of *SLC16A1* (MCT1 gene) mRNA is more consistent across DLBCL cell lines than *SLC16A3* (Supplementary Fig. S1a), is in accordance with MCT1 and MCT4 protein levels in DLBCL patient samples [12]. The chosen cell panel contains examples of cell lines belonging to either the germinal centre B-cell (GCB) or activated B-cell (ABC) cell of origin (COO) classifications, and representatives of the B-cell receptor/proliferation (BCR-DLBCL) or OxPhos-DLBCL subgroups when categorised using the consensus cluster classification (CCC) (Fig. 1a). In addition, the panel contains a heterogeneous set of genetic alterations found clinically in DLBCL, reflecting a diverse range of oncogenic signalling (Supplementary Fig. S1).

The cell panel was initially profiled for expression of MCT1 and 4 protein (Fig. 1b). All cell lines tested expressed MCT1 protein, ranging from high expression in NUDHL1 and OCILY-3 to low expression in Pfeiffer. In contrast, MCT4 protein was only evident in NUDHL1 cells, despite the fact that OCILY-19 expresses comparatively high levels of *SLC16A3* (Fig. 1a, b).

We next examined the effect of AZD3965 or IACS-010759 treatment on intracellular lactate levels or oxygen consumption rate (OCR) respectively. AZD3965 (10 nM) induced a significant accumulation of lactate in all DLBCL cell lines, with the exception of NUDHL1 which expresses MCT4 protein (Fig. 1c). However, the magnitude of lactate accumulation was variable between different cell lines, ranging from a 3-fold increase in Toledo to a 13-fold increase in WSU-DLCL2. In contrast, IACS-010759 treatment led to a dose-dependent reduction in OCR in all cell lines examined (Fig. 1d), irrespective of differences in MCT4 expression, their underlying molecular pathology, or categorisations related to COO or CCC status (Supplementary Fig. S1).

Prior to combination experiments, sensitivity to either AZD3965 and IACS-010759 monotherapy treatment was determined. For AZD3965, in cell lines where GI_50_ values could be determined, values ranged from 11 to 30 nM (Fig. 1e). Although these AZD3965-sensitive DLBCL lines shared relatively similar GI_50_ values, the maximal growth inhibition achieved varied from 98% in WSU-DLCL2 to 60% in RIVA when treated with 1 µM AZD3965. In contrast, GI_50_ values could not be determined for NUDHL1, OCILY-3 or OCILY-19. Whilst the comparative resistance of OCILY-3 and OCILY-19 to AZD3965 treatment cannot be explained by their MCT1 and 4 expression profile, the concentration of lactate accumulated in these lines was less than in the rest of the cell panel, with the exception of MCT4 expressing NUDHL1 (Fig. 1c). Growth inhibitory responses to IACS-010759 treatment were more comparable across the panel, although none of the cell lines tested was highly sensitive to treatment (Fig. 1f). Treatment with 1 µM IACS-010759 for 72 h revealed RIVA to be the most sensitive with 45% growth inhibition being achieved, whilst a maximum of 25% growth inhibition was evident in Farage, Pfeiffer, NUDHL1 and OCILY-3. The growth of Toledo, OCILY-19 and WSU-DLCL2 remained largely unaffected.

**Fig. 2:**
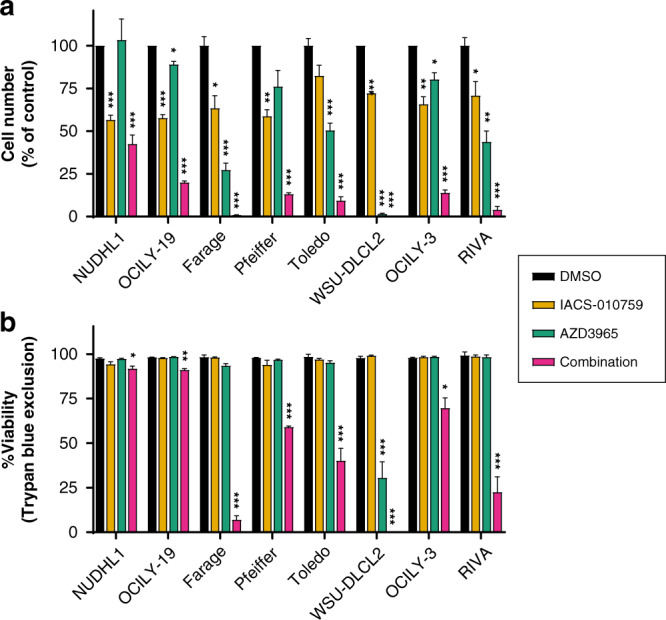
The effect of combining AZD3965 and IACS-010759 treatment in DLBCL cell lines. **a** Cell counts and **b**, percentage viability data (mean + SEM) showing that co-treatment with the Complex I inhibitor IACS-010759 (10 nM) enhances the anti-proliferative effects of AZD3965 (10 nM) and increases cell death (as indicated by Trypan blue staining) in DLBCL cell lines after 72 h (*n* = 4–5). Statistical significance was assessed in relation to vehicle only treatment in a given cell line. Two-tailed *t*-test with multiple comparison correction (Holm–Šídák) highly significant results are indicated ***P ≤ 0.001.

We initially combined AZD3965 and IACS-010759 at 10 nM, given their activity on lactate accumulation and OCR respectively, at this concentration (Fig. 1c, d). Despite the inhibitory effect of AZD3965 on DLBCL growth (Fig. 2a), there was no significant loss in cell viability following monotherapy treatment over a 72 h period (as assessed by Trypan blue staining, Fig. 2b), with the exception of WSU-DLCL2. The latter is one of a small number of tumour cell lines in which evidence of cell death has been reported following treatment with AZD3965 alone [6]. Treatment with IACS-010759 alone also did not induce DLBCL cell death (Fig. 2b). However, the combination of AZD3965 and IACS-010759 was able to induce a substantial decrease in cell number (Fig. 2a) and cell death (Fig. 2b), except in NUDHL1 (MCT4+) and OCILY-19 cells which were maintained at >90% viability. In DLBCL cell lines in which either monotherapy induced minimal cell death (≤6%), the combination resulted in a loss in cell viability that ranged from 29% in OCILY-3 to 93% in Farage. Furthermore, in the WSU-DLCL2 model, where AZD3965 treatment alone induced a 67% loss in cell viability, the combination killed all cells completely.

**Fig. 3:**
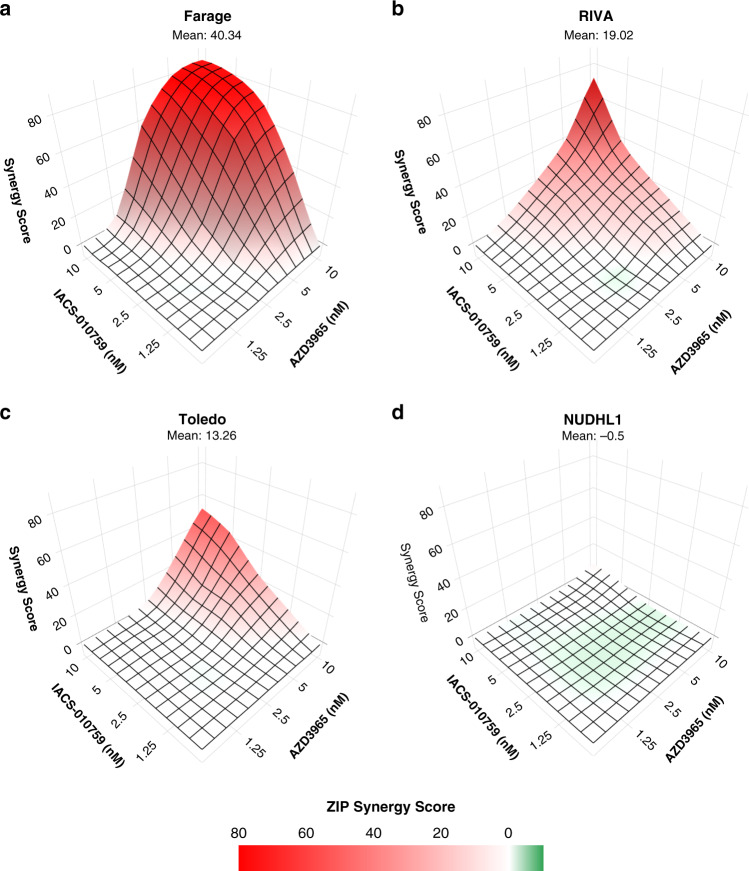
The combination of AZD3965 and IACS-010759 is synergistic in DLBCL cell lines. Synergy was assessed using the Zero Interaction Potency (ZIP) method with SynergyFinder 2.0 in Farage (**a**), RIVA (**b**) and Toledo (**c**) and NUDHL1 (**d**). Each plot represents a single analysis performed on mean viability (72 h) with data derived from 4 to 5 independent experiments.

We chose to characterise the combination interaction in further detail in the Farage, RIVA and Toledo tumour cell lines, where monotherapy treatments had a minimal effect on cell viability but a strong combination effect was evident (Fig. 2b). A concentration range (1.25–10 nM) of each agent was examined alone and in combination to construct a dose matrix, with an analysis of cell viability. This revealed a highly synergistic interaction between the two agents in Farage, RIVA and Toledo (average ZIP synergy score >10) (Fig. 3a–c) [31]. Synergy scores showed a dose-dependent trend, with the highest scores being calculated for Farage. In all three sensitive cell lines, ≥2.5 nM AZD3965 was required to observe synergy with IACS-010759. The addition of IACS-010759, even at the lowest tested concentration (1.25 nM), was sufficient to trigger cell death when combined with 10 nM of AZD3965. The combination did not result in a synergistic interaction in MCT4 expressing NUDHL1 cells (Fig. 3d), or in the GCB-like Burkitt’s lymphoma (BL) cell line BJAB (Supplementary Fig. S2) which was examined as a further example of an MCT4+ tumour cell line.

### In vivo tolerability and efficacy of AZD3965 in combination with IACS-010759

**Fig. 4:**
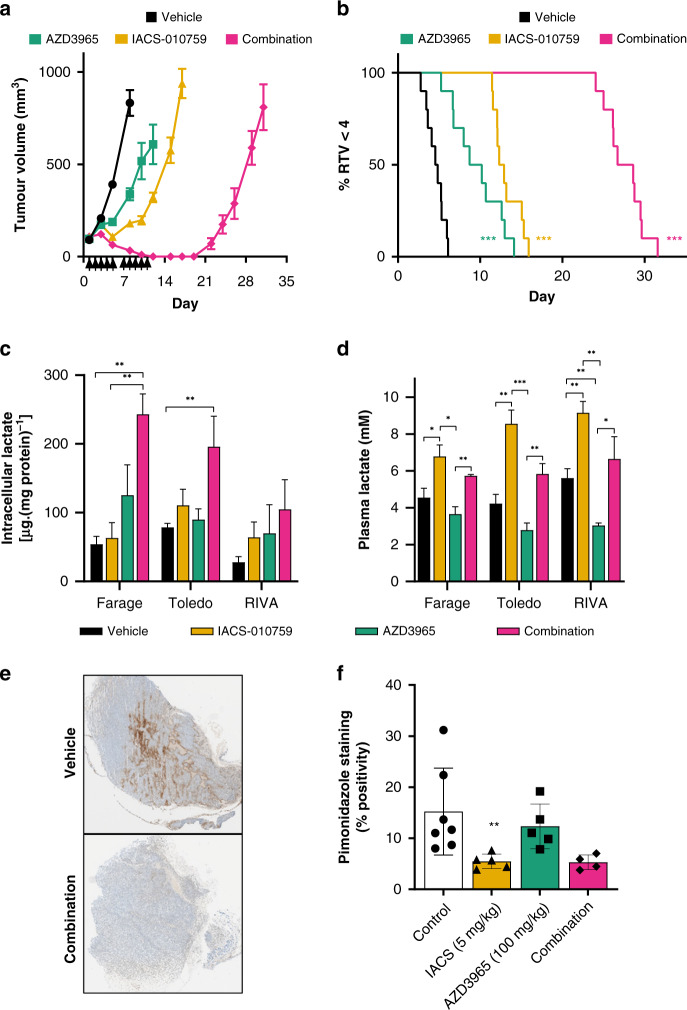
In vivo efficacy of AZD3965 and IACS-010759 in Farage xenografts. **a** Farage cells were injected subcutaneously in NSG mice. Day 1 represents the start of treatment when tumours reached ~100 mm^3^. Mice were divided into four groups (ten mice per group): Vehicle treated (control), AZD3965 (100 mg/kg p.o. BID, 5 days per week), IACS-010759 (5 mg/kg p.o. QD, 5 days per week) or AZD3965 in combination with IACS-010759 under the same schedule as single-agent treatment groups (mean ± SEM). **b** Kaplan–Meier analysis of data generated in study **a**, based on a quadrupling of relative tumour volume (RTV4). Day 1 represents the start of treatments. **c** Concentration of intracellular lactate in DLBCL xenografts (mean + SEM, *n* = 3–5). **d** Concentration of plasma lactate in mice-bearing DLBCL xenografts (mean + SEM, *n* = 3–5). **e** IHC staining for pimonidazole Farage tumours following a single oral treatment with either vehicle or a combination of AZD3965 (100 mg/kg) and IACS-010759 (5 mg/kg). **f** Percentage positivity scores for tumour hypoxia (mean ± SD) in Farage tumours harvested from mice 8 h following a single oral treatment with either vehicle, AZD3965 (100 mg/kg), IACS-010759 (5 mg/kg) or combination therapy.

The metabolic environment in vitro is very homogenous and distinct from the in vivo microenvironment, where regions of the same tumour can differ in the availability of nutrients and the degree of oxygenation [34]. To assess the efficacy of the combination in Farage xenografts we initially selected a dose of 5 mg/kg for IACS-010759 which has been previously studied in in vivo models of AML [35]. The dose of AZD3965 selected (100 mg/kg BID) is sufficient to maintain elevated lactate concentrations in tumour xenografts over a 24-h period and has therefore been widely used for in vivo experiments [6, 8, 36]. Despite potent anti-proliferative effects in vitro, AZD3965 only produced a small growth delayed in Farage xenografts in vivo (Fig. 4a, b). Single-agent treatment with IACS-010759 was also able to delay tumour growth and found to significantly extend the median time to (relative tumour volume x 4) RTV4 by 4 days relative to vehicle-treated mice (*P* < 0.001) (Fig. 4a, b). No regressions were observed in single-agent treatment groups. However, combination treatment (5 days of dosing, followed by a 2-day interval and then a further 5 days of dosing) produced a markedly improved response, with 10/10 complete regressions being observed at the end of the treatment period which were maintained for 8 days before any tumour regrowth was observed. We did not detect any pharmacokinetic interaction between the two compounds when administered concurrently (Supplementary Fig. S3), and the combination treatment appeared to be well tolerated with no significant change in individual bodyweight versus starting body weights (Supplementary Fig. S4).

The pharmacodynamic activity of AZD3965 was investigated in the Farage xenograft model following a single dose of AZD3965 (100 mg/kg). There was an indication of elevated lactate concentrations after 6 h (Fig. 4c), although this did not reach statistical significance (*P* = 0.13). However, whilst single-agent treatment with IACS-010759 (5 mg/kg) did not influence intratumoural lactate concentrations, combined treatment with AZD3965 did induce a significant elevation (*P* = 0.001). This may be a consequence of tumour cells becoming more reliant on glycolytic energy production when treated with IACS-010759, thereby producing greater amounts of lactate which cannot be exported when AZD3965 is co-administered. A similar effect was observed in mice-bearing Toledo xenografts, with combination treatment resulting in significant lactate accumulation (*P* = 0.03, Fig. 4c). Tumour lactate in RIVA xenografts did not change significantly in response to combination treatment (*P* = 0.12), differing from the two other tumour xenograft models and from RIVA cells in vitro (Fig. 4c).

Changes in plasma lactate following Complex I inhibition can indicate target engagement, not only in the tumour, but also in plasma as a consequence of inhibiting Complex I in mouse tissues [37]. IACS-010759 (5 mg/kg) caused a significant increase in plasma lactate in tumour xenograft bearing mice regardless of which model was used (Fig. 4d), with values that were within previously reported values in tumour bearing mice [38]. Interestingly, although not reaching a statistically significant difference (*P* = 0.05–0.25), there was a trend for the IACS-010759 elevated plasma lactate levels to be reduced when mice were treated concomitantly with AZD3965; the plasma lactate concentrations measured within each combination group being not significantly different to those measured in corresponding vehicle-treated mice. We also examined tumour hypoxia by utilising pimonidazole and immunohistochemistry, a reduction of which is recognised as a biomarker for the inhibition of oxidative phosphorylation as tumour oxygen consumption falls [39]. Treatment with a single dose of IACS-010759 (5 mg/kg) was found to reduce tumour hypoxia significantly, 4 h after drug administration (Fig. 4e, f). This was not affected by co-administration with AZD3965 (100 mg/kg) (Fig. 4f).

### Examining an IACS-010759 dose response in vivo

The concentrations of metabolic inhibitors examined preclinically can greatly exceed those that are tolerated in patients and must be considered for appropriate translation to the clinic [40]. Previous preclinical studies that examined an alternative inhibitor, BAY 87-2243, despite tolerability in mouse models, led to unexpected clinical toxicities which were observed during Phase I studies for this agent [41, 42].

Although both targets have potential mechanistic toxicities, most cell types are not reliant on MCT1 for lactate transport, including highly glycolytic cell types, such as fast-twitch muscle fibres or astrocytes, which co-express MCT4. This is also true for most major organs which express both MCT1 and MCT4 including the heart, liver, kidneys and lungs [43]. In contrast, the level of Complex I inhibition requires careful titration to avoid systemic toxicities due to the more ubiquitous dependency of normal tissues on OXPHOS. We, therefore, assessed both the efficacy and pharmacokinetics of IACS-010759 in mice at lower doses.

**Fig. 5:**
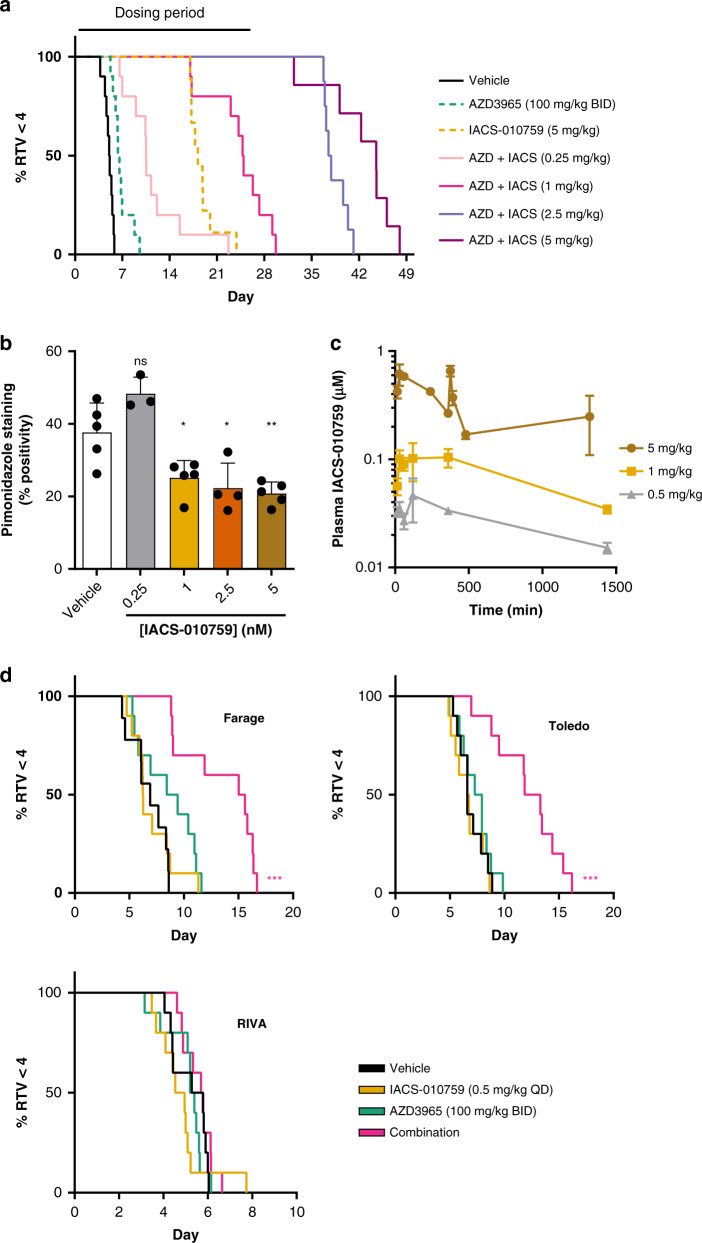
In vivo synergy between AZD3965 and IACS-010759. **a** NSG mice-bearing Farage xenografts were divided into 7 groups (ten mice per group) and treated for a period of 4 weeks: Vehicle treated (control), AZD3965 (100 mg/kg p.o. BID, 5 days per week, IACS-010759 (5 mg/kg p.o. QD, 5 days per week) or AZD3965 in combination with IACS-010759 (0.25, 1, 2.5 or 5 mg/kg) under the same schedule as single-agent treatment groups. Significant differences in time to RTV4 control were observed between all treatment groups and vehicle (*P* < 0.0001). **b** IHC staining for pimonidazole of Farage tumours harvested from mice following treatment with vehicle or IACS-010759 at 8 h. Significance versus vehicle-treated values assessed using a two-tailed *t*-test. **c** Plasma concentration of IACS-010759 in NSG mice, data points show mean ± SEM of three samples per timepoint. **d** Response of Farage, Toledo or RIVA xenografts to treatment. Kaplan–Meier plots for mice treated from day 1 (ten mice per group) based on RTV4: Vehicle treated (control), AZD3965 (100 mg/kg p.o. BID, 5 days per week), IACS-010759 (0.5 mg/kg p.o. QD, 5 days per week) or AZD3965 in combination with IACS-010759 (0.5 mg/kg). Kaplan–Meier significance assessed by the Log-rank test followed by the Holm–Šídák method to correct for multiple comparisons.

AZD3965 combined with a range of IACS-010759 doses (0.25–5 mg/kg) revealed a clear dose-dependent response in mice-bearing Farage xenografts (Fig. 5a). All treatment groups showed a significant benefit in time to RTV4 versus vehicle (*P* < 0.0001). However, the effects were most pronounced in the combination groups examining IACS-010759 at 1 mg/kg and greater, with all groups extending median survival time over single-agent treatment IACS-010759 (5 mg/kg) by 6 to 26 days. IACS-010759 also significantly reduced the number of pimonidazole positive tumour cells at doses of ≥1 mg/kg, reflecting diminished tumour hypoxia as a consequence of drug treatment (Fig. 5b).

Pharmacokinetic analysis revealed exposure levels in vivo following a 5 mg/kg dose were significantly higher than those which can be achieved safely clinically (Fig. 5c) [44]. C_max_ levels in vivo were around 500 nM and occurred rapidly (<2 h) after oral administration. In contrast, a single 0.5 mg/kg dose of IACS-010759 yielded a C_max_ value of 46 nM and a C_min_ of 15 nM which we estimate is a closer approximation of steady-state plasma concentrations of around 20 nM that have been attained clinically [44]. We, therefore, examined this dose, which is tenfold lower than that typically used in preclinical studies, in combination with AZD3965 in Farage, Toledo and RIVA DLBCL xenografts [18, 21, 35], Single-agent treatment had no significant effect on Farage tumour growth. This was in contrast with our initial efficacy study where AZD3965 demonstrated a small but significant increase in time to reach RTV4 in the same model. The growth of vehicle-treated tumours was slightly slower in this experiment, as evidenced by an increase in the mean time to reach an RTV4, from 4.6 days in our initial Farage study to 6.8 days (*P* < 0.003) in our third study, which may have influenced the response to AZD3965 alone. Combining AZD3965 (100 mg/kg BID) and IACS-010759 (0.5 mg/kg QD) revealed a highly significant improvement in time to reach RTV4 (*P* < 0.01) in both Farage and Toledo tumour models versus vehicle or either monotherapy (Fig. 5d). However, under these conditions, no tumour regressions were observed. Despite potent combination effects being observed in vitro, neither the single agent or combination treatment induced a response in RIVA xenografts, with all tumours reaching RTV4 within 10 days (Fig. 5d), potentially reflecting the aforementioned inability of the combination to induce a significant increase in RIVA tumour lactate (Fig. 4c).

### Altered metabolic phenotype of cells exposed to IACS-010759 and AZD3965

**Fig. 6:**
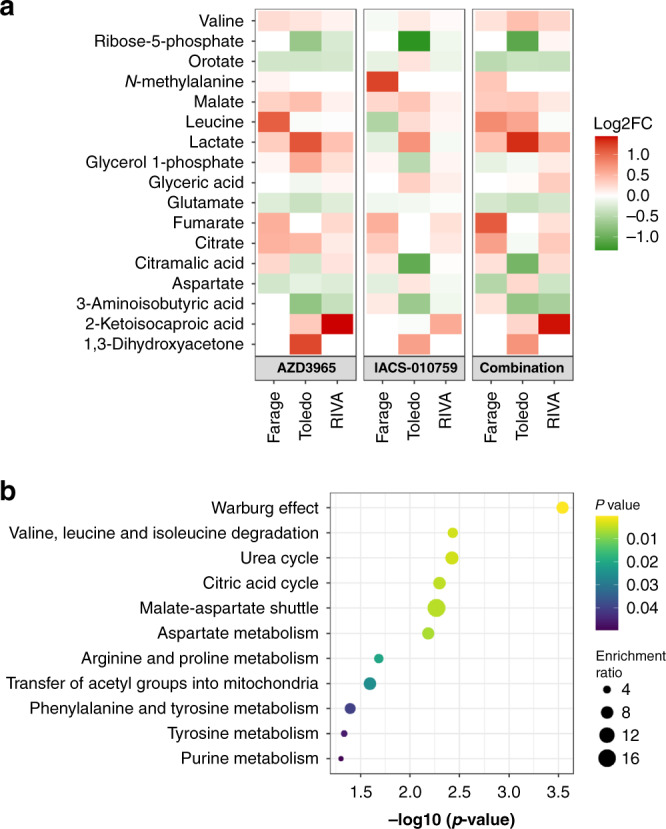
Metabolic response to AZD3965 in combination with IACS-010759 in vivo. NSG mice-bearing subcutaneous DLBCL xenografts were treated with AZD3965 (100 mg/kg) ± IACS-010759 (0.5 mg/kg) or vehicle and tumours collected 6 h following AZD3965, 4 h post IACS-010759. **a** Metabolite abundance expressed as log_2_ fold change versus vehicle-treated controls. Metabolites were significantly altered in at least one group as assessed by one-way ANOVA with Tukey correction for multiple comparisons. **b** pathway enrichment score using significantly altered metabolites observed in 1 or more DLBCL xenografts.

The disparate responses of the different DLBCL xenografts to combination treatment in vivo may conceivably be attributable to different metabolic adaptations. To examine this, we determined the metabolic consequences of combining AZD3965 (100 mg/kg) and IACS-010759 (0.5 mg/kg) in vivo by performing GC-MS analysis on DLBCL xenograft samples following acute treatment. An acute timepoint was chosen to aid the deconvolution of the primary metabolic pathways potentially responsible for the disparate responses between cell lines and to reduce the potential for metabolic adaptation. This also helped to limit the possible impact of varying tumour size between treatment groups on tumour metabolism. Peak intensities for each metabolite were compared to samples from vehicle-treated animals to provide log_2_ fold-change values (Fig. 6a). Treatment with AZD3965, either alone or in combination with IACS-010759, significantly decreased (*P* < 0.05) levels of glutamate in Farage, RIVA and Toledo xenografts which is consistent with previous data in BL xenografts [12, 45]. Consistent with this effect, in independent studies, AZD3965 and IACS-010759 have been combined with the glutaminase inhibitor BPTES, and increased in vitro sensitivity observed in BL and Mantle cell lymphoma cell lines respectively [6, 18]. Relatively few altered metabolites were observed following low dose (0.5 mg/kg) single-agent IACS-010759 treatment.

Pathway analysis indicated significant enrichment for metabolites involved in the Warburg Effect including lactate but also citrate and aspartate following combination treatment (SMP0000654) (Fig. 6b). Interestingly, lactate was significantly elevated in RIVA xenografts after combination treatment. Therefore, PD biomarkers like tumour lactate can show target engagement but do not necessarily function as markers of efficacy. RIVA did not appear dramatically different in the acute metabolite profile following combination treatment in comparison to sensitive tumour models, apart from a strong increase in α-ketoisocaproate, an intermediate of l-leucine metabolism and an MCT1 substrate [46].

## Discussion

Inhibition of MCT1, represents a novel approach to target tumour metabolism in tumours that lack MCT4. A tumour selective effect is derived from the expression of MCT4 in normal glycolytic tissues, which can enable a compensatory efflux of lactate to circumvent the consequences of MCT1 blockade. This therapeutic strategy may be relevant to the treatment of highly glycolytic lymphoma malignancies that lack MCT4, particularly if the effect can be exploited further using an appropriate combination strategy. In this study, we evaluated a combination of MCT1 inhibition and Complex I inhibition, to simultaneously target both the glycolytic and OXPHOS metabolic characteristics of DLBCL tumour cells.

Our data indicate that whilst AZD3965 monotherapy treatment does lead to a significant accumulation of lactate in DLBCL cells without MCT4 protein, the concentration of intracellular lactate attained, and the resultant phenotypic consequences, were highly variable between individual cell lines. Near-complete growth inhibition was evident in WSU-DLCL2, intermediate growth inhibitory responses were observed in Pfeiffer, RIVA, Toledo and Farage, and OCILY-19 and OCILY-3 were found to be comparatively resistant to treatment. These responses broadly correlate with the magnitude of lactate accumulated following AZD3965 treatment, in that the highest lactate concentrations were evident in WSU-DLCL2 (291 ± 67 µg/mg protein), and the lowest (29 µg/mg protein) in OCILY-19 and OCILY-3, potentially reflecting differences in their reliance upon glycolysis. The response to IACS-010759 monotherapy treatment was more consistent but only a partial growth inhibition was induced in each DLBCL cell line. IACS-010759 activity was not dependent upon the CCC classification of cell lines, Toledo being an OxPhos-DLBCL cell line that proved the most refractory to IACS-010759 treatment.

With the exception of WSU-DLCL2, AZD3965 or IACS-010759 monotherapy activity involved a reduction in cellular proliferation. However, the combination of both compounds resulted in 29–100% tumour cell killing in six out of seven MCT1+/MCT4− DLBCL lines, confirming that the simultaneous targeting of two key metabolic pathways can deliver a highly pronounced therapeutic effect. Using the Farage DLBCL xenograft model, it was also possible to demonstrate that a combination effect could be achieved in vivo, even after a relatively short treatment period, using a 5 mg/kg dose of IACS-010759 that has been examined previously in preclinical studies [17, 18, 21]. When a 0.5 mg/kg dose of IACS-010759 was selected as one that would generate plasma exposure levels nearer to those achieved clinically (~20 nM), an enhanced combination effect was still evident in two of three DLBCL xenograft models, resulting in a significant tumour growth delay when there was little or no evidence of activity with either monotherapy alone [44]. This is encouraging given the comparatively low dose of IACS-010759 used and that AZD3965 (100 mg/kg BID) treatment was inactive in the DLBCL xenograft models examined. In contrast, a clinical study examining AZD3965 monotherapy treatment of 11 DLBCL patients, observed evidence of stable disease in one patient and a prolonged >50% partial response in another [47]. The potential to augment AZD3965 clinical activity significantly through combined treatment with IACS-010759, therefore, represents an attractive concept. It is also conceivable that a higher maximum tolerated dose of IACS-010759 could be co-administered, perhaps using an episodic dosing schedule to transiently maximise the combination effect in tumour cells, something which requires further investigation.

That RIVA xenografts did not respond to combination treatment, would not have been anticipated from the in vitro RIVA cell line studies in which strong combination effects were also observed. The accumulation of lactate in RIVA xenografts following combination treatment failed to reach statistical significance. Our metabolomics data also revealed an increase in α-ketoisocaproate in RIVA xenografts, which may indicate that leucine can act as an energetic substrate in this model through its conversion to α-ketoisocaproate by branched-chain amino acid aminotransferase (BCAT). This metabolite can be converted to isovaleryl-CoA and fed into the TCA cycle to produce ATP, thus protecting the cell from death [48, 49]. These observations may partly explain why RIVA xenografts are refractory to the combination of AZD3965 and IACS-010759, and further highlight challenges in being able to accurately predict the metabolic dependencies of tumours in vivo from in vitro studies.

We observed a significantly increased plasma lactate concentration in mice following administration of a large (5 mg/kg) dose of IACS-010759, something that has also been reported in patients and which, although not associated with acidosis, may be responsible for incidences of peripheral neuropathy [44]. Although not reaching statistical significance, there was a trend for combined AZD3965 administration to lower the elevated plasma lactate concentrations induced by IACS-010759 treatment alone. This suggests that a combination of the two agents may not only have the potential to lead to greater efficacy but may also enhance tolerability.

Collectively, these studies exemplify the concept of dual-metabolic targeting and show that a potential therapeutic window for the combination of AZD3965 and IACS-010759 exists in DLBCL, thereby providing a rationale for examining the efficacy of these agents clinically.

## Supplementary information

Simultaneous targeting of glycolysis and oxidative phosphorylation as a therapeutic strategy to treat diffuse large B-cell lymphoma: Supplemental Data

Checklist

## Data Availability

All data required to evaluate the findings of this study is available in the manuscript and online Supplementary Materials.
