# Supplementary material for: Simultaneous targeting of glycolysis and oxidative phosphorylation as a therapeutic strategy to treat diffuse large B-cell lymphoma
Source: Br J Cancer. 2022 May 26;127(5):937–47. doi: 10.1038/s41416-022-01848-w (PMC9428179; doi:10.1038/s41416-022-01848-w)
Supplement: Supplementary file 1 — Simultaneous targeting of glycolysis and oxidative phosphorylation as a therapeutic strategy to treat diffuse large B-cell lymphoma: Supplemental Data [file 41416_2022_1848_MOESM1_ESM.pdf]

Simultaneous targeting of glycolysis and oxidative phosphorylation as a therapeutic strategy to treat diffuse large B-cell lymphoma

Supplemental Data

Richard A. Noble[1], Huw Thomas[1], Yan Zhao[1], Lili Herendi[2], Rachel Howarth[3], Ilaria Dragoni[4], Hector C. Keun[2], Christopher P. Vellano[5], Joseph R. Marszalek[5] and Stephen R. Wedge[1].

Supplementary figures

Figure S1.

a Cell of origin (COO) subtype[1, 2], consensus cluster classification (CCC)[3-5] and molecular characteristics of cell lines used in the study. CCLE data[6] on mutation, copy number and structural variants was collected from cBioPortal[7]. No copy number variation data was available for Farage. **b** *SLC16A1* (*MCT1*) and *SLC16A3* (MCT4) RNA expression in DLBCL cell lines (n=26). RNA sequencing data (expressed as log_2[transcripts per million + 1]) for DLBCL cell lines was downloaded from the Broad Institute Dependency Map Portal (https://depmap.org) (Expression 21Q4 Public).

Figure S2.

a Cell counts and **b,** percentage viability data (mean + SEM) showing co-treatment with the Complex I inhibitor IACS-010759 (10 nM) and AZD3965 (10 nM) does not increases cell death (as indicated by Trypan blue staining) in BJAB after 72 hours (*n* = 4). **c** dose-response matrices between AZD3965 and IACS-010759 in BJAB. Synergy was assessed using the Zero Interaction Potency (ZIP) method with SynergyFinder 2.0[8]. Each plot represents a single analysis performed on mean viability (72 hours) data taken from 4 independent experiments.

Figure S3.

Plasma pharmacokinetics of AZD3965 and IACS-010759 following oral dosing to mice do not indicate an interaction between the two. **a** PK profiles of AZD3965 (100 mg/kg BID) over 8 hours ± co-administration with IACS-010759 where plasma concentrations were collected at 5 hours. **b** Plasma concentrations of IACS-010759 at 3 and 24 hours when administered as single-agent or in combination with AZD3965 (mean ± SEM). **c** PK parameters for single agent IACS-010759 5, 1 or 0.5 mg/kg.

Figure S4.

Body weights (Mean ± SD) during efficacy studies in Farage tumour bearing mice receiving AZD3965 (100 mg/kg BID), IACS-010759 (5 mg/kg), the combination, or a matched vehicle control.

Supplementary references

1. Hicks S. W., Tarantelli, C., Wilhem, A., Gaudio, E., Li, M., Arribas, A. J. *et al*. The novel CD19-targeting antibody-drug conjugate huB4-DGN462 shows improved anti-tumour activity compared to SAR3419 in CD19-positive lymphoma and leukemia models. *Haematologica* **104**, 1633-1639 (2019).

2. Bethge, N., Honne, H., Hilden, V., Trøen, G., Eknæs, M., Liestøl, K. *et al*. Identification of highly methylated genes across various types of B-cell non-hodgkin lymphoma. *PLoS One* **8**, e79602-e79602 (2013).

3. Polo, J. M., Juszczynski, P., Monti, S., Cerchietti, L., Ye, K., Greally, J. M. *et al*. Transcriptional signature with differential expression of BCL6 target genes accurately identifies BCL6-dependent diffuse large B cell lymphomas. *Proc Natl Acad Sci U S A* **104**, 3207-3212 (2007).

4. Polo, J. M., Juszczynski, P., Monti, S., Cerchietti, L., Ye, K., Greally, J. M. *et al*. Study of the antilymphoma activity of pracinostat reveals different sensitivities of DLBCL cells to HDAC inhibitors. *Blood Adv* **5**, 2467-2480 (2021).

5. Chen, L., Monti, S., Juszczynski, P., Daley, J., Chen, W., Witzig, T. E. *et al*. SYK-dependent tonic B-cell receptor signaling is a rational treatment target in diffuse large B-cell lymphoma. *Blood* **111**, 2230-2237 (2008).

6. Chen, L., Monti, S., Juszczynski, P., Daley, J., Chen, W., Witzig, T. E. *et al*. Next-generation characterization of the Cancer Cell Line Encyclopedia. *Nature* **569**, 503-508 (2019).

7. Gao, J., Aksoy, B. A., Dogrusoz, U., Dresdner, G., Gross, B., Sumer, S. O. *et al*. Integrative analysis of complex cancer genomics and clinical profiles using the cBioPortal. *Sci Signal* **6**, pl1 (2013).

8. Ianevski A., Giri A. K., Aittokallio T. SynergyFinder 2.0: visual analytics of multi-drug combination synergies. *Nucleic Acids Research* **48**, W488-W493 (2020).

Figure S1

a

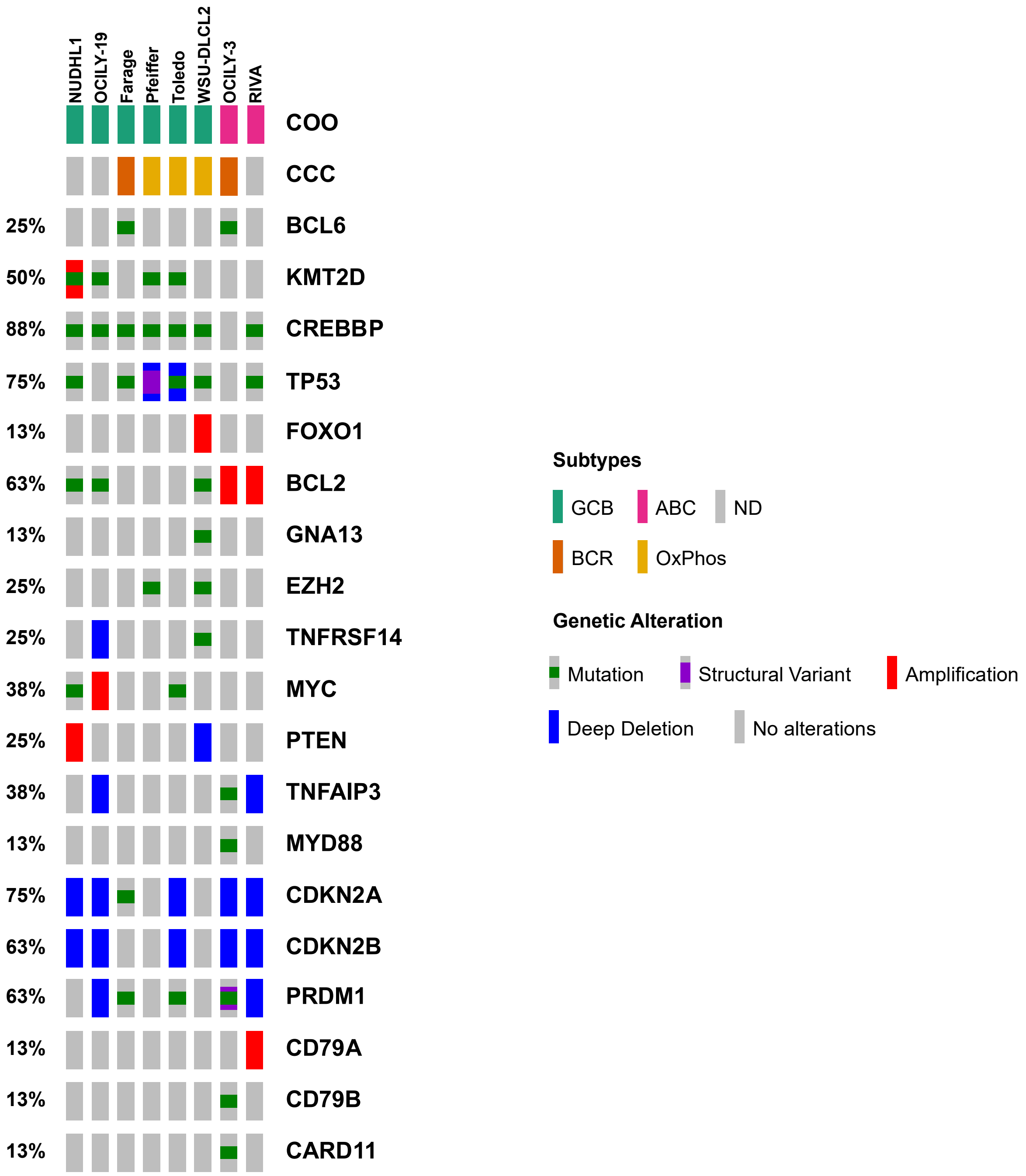
NUDHL1
OCILY-19
Farage
Pfeiffer
Toledo
WSU-DLCL2
OCILY-3
RIVA
COO
CCC
25% BCL6
50% KMT2D
88% CREBBP
75% TP53
13% FOXO1
63% BCL2
13% GNA13
25% EZH2
25% TNFRSF14
38% MYC
25% PTEN
38% TNFAIP3
13% MYD88
75% CDKN2A
63% CDKN2B
63% PRDM1
13% CD79A
13% CD79B
13% CARD11
Subtypes
GCB
ABC
ND
BCR
OxPhos
Genetic Alteration
Mutation
Structural Variant
Amplification
Deep Deletion
No alterations

b

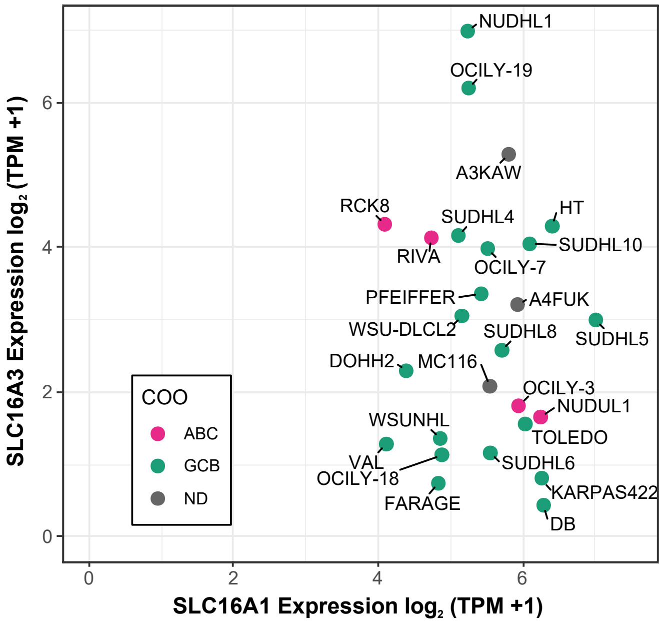
SLC16A3 Expression log₂ (TPM +1)
SLC16A1 Expression log₂ (TPM +1)
NUDHL1
OCILY-19
A3KAW
RCK8
SUDHL4
HT
RIVA
SUDHL10
OCILY-7
PFEIFFER
A4FUK
WSU-DLCL2
SUDHL8
SUDHL5
DOHH2
MC116
OCILY-3
NUDUL1
WSUNHL
TOLEDO
VAL
OCILY-18
SUDHL6
FARAGE
KARPAS422
DB
COO
ABC
GCB
ND
0
2
4
6

Figure S2

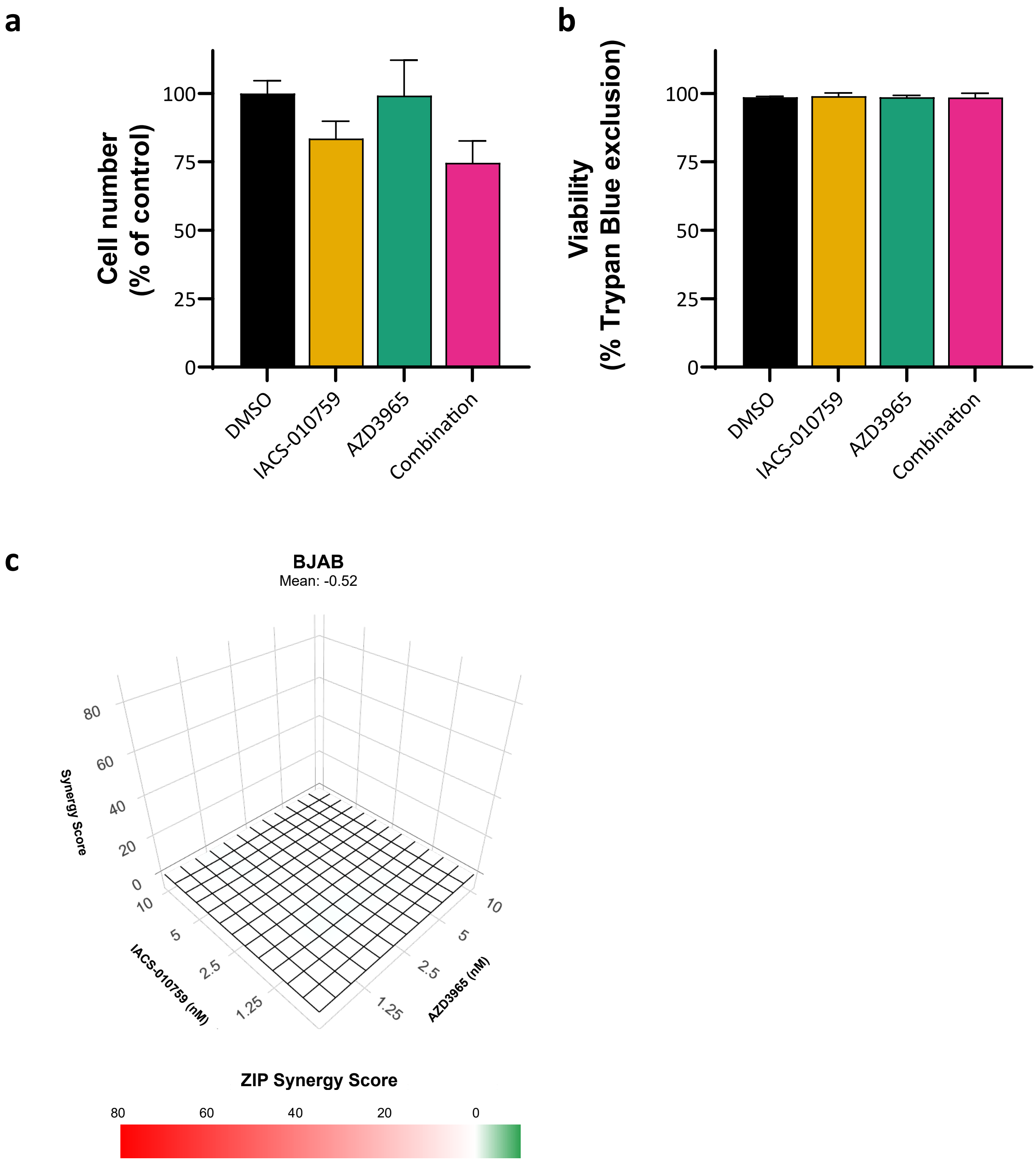
a
Cell number
(% of control)
100
75
50
25
0
DMSO
IACS-010759
AZD3965
Combination
b
Viability
(% Trypan Blue exclusion)
100
75
50
25
0
DMSO
IACS-010759
AZD3965
Combination
c
BJAB
Mean: -0.52
Synergy Score
80
60
40
20
0
10
5
2.5
1.25
IACS-010759 (nM)
AZD3965 (nM)
ZIP Synergy Score
80
60
40
20
0

Figure S3

a

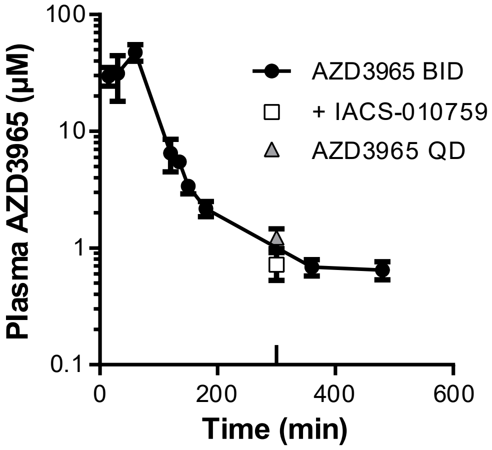

b

Time (h)	[IACS-010759]$_{plasma}$ (μM)	[IACS-010759]$_{plasma}$ coadministered with AZD3965 (μM)
**3**	0.35 ± 0.030 (n=5)	0.36 ± 0.032 (n=5)
**24**	0.024 ± 0.002 (n=5)	0.035 ± 0.008 (n=5)

c

Parameter	IACS-010759 Plasma PK		
Dose (mg/kg) p.o	**5**	**1**	**0.5**
AUC inf (μg/ml.min)	300	90	35
AUC last (μg/ml.min)	225	66	24
C Max (μg/ml)	0.37	0.06	0.028
T Max (min)	30	120	120
Half Life α (min)	349.0	791.1	854.8
Clearance$_{app}$ (ml/min/kg)	16.7	11.1	14.2

Figure S4

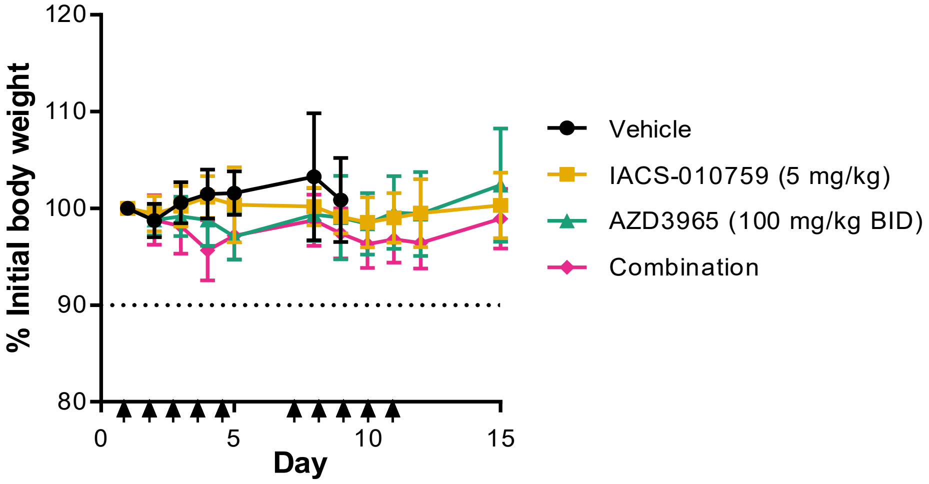
120
110
100
90
80
% Initial body weight
0
5
10
15
Day
Vehicle
IACS-010759 (5 mg/kg)
AZD3965 (100 mg/kg BID)
Combination
